# Metastasis-related genomic aberrations and evolutionary trajectory in uveal melanoma

**DOI:** 10.1038/s12276-026-01750-y

**Published:** 2026-06-17

**Authors:** Chang Hyun Nam, Yong Joon Kim, Jeonghwan Youk, Hyo Song Park, Young Seok Ju, Christopher Seungkyu Lee

**Affiliations:** 1 Graduate School of Medical Science and Engineering, Korea Advanced Institute of Science and Technology, Daejeon, Republic of Korea; 2 Institute of Vision Research, Department of Ophthalmology, Severance Hospital, Yonsei University College of Medicine, Seoul, Republic of Korea; 3 Department of Internal Medicine, Seoul National University Hospital, Seoul, Republic of Korea; 4 Department of Ophthalmology, College of Medicine, Soonchunhyang University, Cheonan, Republic of Korea; 5 Department of Ophthalmology, Soonchunhyang University Hospital Bucheon, Bucheon, Republic of Korea

**Keywords:** Eye cancer, Cancer genomics, Oncogenes, Bioinformatics, Medical genomics

## Abstract

Uveal melanoma (UM) is the most common primary intraocular cancer, characterized by genetic variations associated with metastasis-related mortality. Current prognostic classifications primarily rely on large-scale copy number alterations without incorporating more precise genomic aberrations. We present a comprehensive analysis of whole-genome sequences from 40 primary UMs and matched normal tissues, revealing a complete landscape of somatic aberrations, including loss of heterozygosity and structural variations. It enabled the identification of two novel metastatic markers: *BAP1* aberrations (including loss of heterozygosity and structural variation-associated truncations, which were not revealed by previous targeted approaches) and 1q gains, thereby enhancing current molecular stratification for metastatic risk. Mutation timing analysis revealed that prevalent copy gains were often acquired early in life, several decades before tumour diagnosis. Notably, early 6p gain was frequently exclusive of *BAP1* aberrations and associated with a favourable prognosis, suggesting that metastatic potential in UM may be established early during tumorigenesis. Collectively, our findings provide molecular changes contributing to tumorigenesis and metastatic progression in UM and offer potential biomarkers for early detection and prognosis prediction.

## Novel Markers Enhance Prognostic Prediction in Uveal Melanoma

Uveal melanoma (UM) is the most common primary intraocular cancer in adults, with a high risk of metastasis and limited treatment options. This study explores the genomic landscape of UM using whole-genome sequencing to identify key mutations linked to metastasis. The authors analysed 40 UM samples in the discovery phase, revealing that *BAP1* gene aberrations and 1q gain are significant predictors of metastatic progression. These findings suggest a new genomic classification system that improves prognostic prediction compared with the existing Cancer Genome Atlas classification. The study also highlights that copy number gains, particularly at 6p, 8q and 1q, often occur early in life, potentially shaping later metastatic potential. This research enhances understanding of the genomic evolution of UM, offering insights for early detection and improved prognostication, warranting further mechanistic investigation of copy number alterations.

This summary was initially drafted using artificial intelligence, then revised and fact-checked by the author.

## Introduction

Uveal melanoma (UM) is the most common primary intraocular malignancy in adults^1^. Despite successful local treatment, up to 50% of patients eventually develop metastasis^2^ and typically experience mortality within 12 months of detection^3^, primarily because of the lack of effective systemic therapies. The unmet need for UM treatment highlights the importance of investigating the mechanisms underlying metastatic progression and identifying molecular markers for prognosis prediction.

Previous studies revealed genetic variations related to tumour development and metastasis in UM^1^. Nearly all UM tumours harbour mutually exclusive hotspot mutations affecting genes in the Gα pathway, including *GNAQ* and *GNA11*^4,5^, suggesting that the activation of the pathway might be linked to UM initiation. Subsequent genomic mutations further drive tumorigenesis and even lead to metastatic diseases, as evidenced by their association with poor prognosis. For example, the loss of one copy of chromosome 3, known as monosomy 3, and loss-of-function mutations in the *BAP1* gene (located on chromosome 3p) correlate strongly with an increased risk of metastasis and reduced survival^6,7^. Furthermore, *SF3B1* mutations and 8q gain are related to a poor prognosis, whereas *EIF1AX* mutations and 6p gain are associated with a favourable prognosis^8–10^. Overall, a prognostic molecular classification was introduced based on 80 tumour exome sequences from The Cancer Genome Atlas (TCGA)^11,12^, which categorizes UM into four distinct subtypes, primarily characterized by arm-level copy number alterations (CNAs), i.e. monosomy 3 and 8q gain. The prognostic implications of this classification have been validated through long-term follow-up studies^13,14^.

Recent whole-genome sequencing (WGS) studies have revealed a low mutational burden and a limited impact of ultraviolet light on mutagenesis in UM^15–17^. However, a comprehensive investigation of genomic aberrations, including structural variations (SVs) and loss of heterozygosity (LOH), remains to be thoroughly conducted, particularly regarding their prognostic implications. In addition, integrative analysis of CNAs and point mutations can elucidate the genomic evolution of cancer, as demonstrated in clear-cell renal-cell carcinomas and lung adenocarcinomas^18,19^. However, this approach has yet to be explored in UM. Furthermore, the genetic landscape of UM in Asian patients remains significantly underrepresented, largely because of its lower incidence in this population^1^, highlighting the need for further studies to address this gap and provide population-specific insights.

Here, we comprehensively illustrate the mutational landscape of UM through WGS and estimate the impact of genomic alterations on metastatic potential. Our findings demonstrate a strong and independent association of *BAP1* aberrations and 1q gain with metastatic progression in primary UM, suggesting a novel genomic classification that offers enhanced prognosis prediction compared with the previous TCGA classification. Furthermore, we estimate that prevalent copy number gains, some of which are linked to metastatic recurrence, frequently emerge during the early decades of life, often decades before clinical diagnosis, contributing to the early establishment of metastatic potentials in UM tumours.

## Materials and Methods

### Collection of uveal melanoma samples

This study was conducted prospectively, enrolling participants from June 2018 to July 2021, with written informed consent. Adult patients (age ≥ 20) presenting with suspected uveal melanoma and deemed eligible for biopsy were included in the study. Tumour samples were obtained through either trans-scleral or trans-retinal aspiration biopsy based on tumour location and subsequently underwent pathological confirmation of uveal melanoma as part of standard clinical practice. Patients who were unable to provide informed consent or from whom sufficient samples could not be obtained were excluded from the study. The disease extent was assessed using slit-lamp biomicroscopy, fundus examination, fundus photography, fluorescein angiography, indocyanine green angiography, ultrasonography, orbital MRI, abdominal MRI and PET-CT. Clinical data, including age, sex, tumour size, tumour location, treatment modality, metastasis and mortality, were collected in compliance with the study protocol. The study protocol was reviewed and approved by the institutional review boards of Yonsei University Health System (IRB No: 4-2018-0388) and Korea Advanced Institute of Science and Technology (IRB No: KH2023-160).

### Preparation of validation dataset

To validate our findings, we analysed the whole-genome sequences of uveal melanomas studied by the International Cancer Genome Consortium (ICGC)^16^. Of the 103 UM tumours in this dataset, we included 75 tumours with characteristics comparable to our cohort, including choroidal location, absence of germline *MBD4* mutations, tumour purity ≥ 0.15 and available relapse-free survival data.

### Library preparation and whole-genome sequencing

Genomic DNA was extracted from tumour and matched blood samples using the Gentra Puregene Tissue Kit (Qiagen) and the MG Blood Genomic DNA Extraction Kit (MGmed), respectively. The DNA libraries were prepared with the TruSeq DNA PCR-Free (Illumina), TruSeq DNA Nano (Illumina) and Accel-NGS 2S Plus DNA Library Kit (Swift), depending on the DNA amount obtained from the samples. The DNA libraries were sequenced using the Illumina HiSeq X Ten platform.

### Variant calling and filtering of whole-genome sequencing data

Sequenced reads were aligned to the human reference genome (GRCh37) using the BWA-MEM algorithm^20^. Duplicated reads were eliminated using Picard (available at http://broadinstitute.github.io/picard). Further processing, including indel realignment and base quality score recalibration, was conducted via GATK^21^. Single-nucleotide variants (SNVs) and short indels were detected as previously described^19^ with slight modification. Briefly, base substitutions and short indels were called using Mutect2^21^, Varscan2^22^ and Strelka2^23^ and merged to obtain a union set. Then, we included variants with the following features: 1% or less variant allele fraction (VAF) in the panel of normals, fewer than one variant read in the matched normal, three or more variant reads with higher than 5% of VAF in tumour samples, a low proportion (< 50%) of clipped or discordant reads among variant reads, fewer than three median mismatched bases in variant reads, variant reads identified in both strand and orientation, and locations farther than 10% of the read length from both read ends. Known driver mutations common in uveal melanoma^1^ were rescued to ensure sensitive detection of driver mutations.

### Mutational signature analysis

We utilized SigProfiler^24^ to estimate the contributions of signatures in tumour samples. In brief, we decomposed the mutational profiles of 40 primary UM tumours using COSMIC mutational signature version 3.3 (available at https://cancer.sanger.ac.uk/cosmic/signatures). We then examined the fitting results by assessing the concordance between the observed mutational profiles and those reconstructed from the inferred signatures, and excluded signatures that were likely to represent technical artefacts arising from overfitting. Finally, SBS1, SBS2, SBS5, SBS13, SBS18 and SBS40 were selected to estimate the relative contributions of mutational signatures in each sample.

### Clustered mutation analysis

We used SigProfilerClusters^25^ to identify clustered somatic point mutations and trace the evidence of APOBEC-related mutagenesis. Briefly, it calculates the sample-specific cutoff value of intermutational distance (IMD) for clustered mutations based on the mutational burden and patterns. Then, the SNVs are classified into non-clustered SNVs, doublet-base substitutions, multi-base substitutions, omikli and kataegis. The latter two categories are defined when IMD is less than the sample-specific threshold and greater than 1, with the number of mutations being two or three and more than four, respectively.

### Estimation of purity, ploidy and allele-specific copy numbers

We utilized the Sequenza^26^ and Battenberg^27^ algorithms to estimate tumour purity, ploidy and allele-specific copy numbers. Two different algorithms primarily generated consistent estimates of purity and ploidy. However, when the solutions were discordant, we selected the solution based on the copy number profiles. Specifically, we assumed that the solution that best explains the distribution of depth ratio and B allele frequency with the fewest ploidy and subclonal CNAs was the most appropriate. We ran the Sequenza algorithm with refined purity and ploidy values if the Sequenza output was not selected. Segmentation results were smoothed by incorporating small segments (< 1 Mb) into an adjacent one to remove false-positive CNAs. Then, we merged the segmentation borders and SV breakpoints to obtain the final segmentation results. Last, we re-ran the Sequenza algorithm with the refined purity, ploidy and segmentation results to generate the final CNA output. We determined the whole-genome duplication (WGD) status based on ploidy estimates and the fraction of genomes exhibiting LOH, as previously described^28^.

### Analysis of structural variations

SVs were called using DELLY^29^ and SvABA^30^ and merged to obtain a union set. SV candidates were annotated using GREMLIN (https://github.com/phansol/gremlin) and filtered as previously described^19^ with slight modification. Briefly, we removed calls with the following features: presence in the panel of normals or matched normal, insufficient number of supporting read pairs (fewer than five read pairs with no supporting SA tag or fewer than three read pairs with at least one supporting SA tag), and many discordant reads in matched blood samples. Then, we visually examined all SV candidates using Integrative Genomics Viewer^31^ to remove remaining false-positive calls and rescue false-negative events that were located near the breakpoints or regions with copy number changes. Next, we clustered SVs when breakpoints between two different SVs were closely located (less than 5 Mb) and defined complex genomic rearrangement (CGR) as a cluster that contains more than ten breakpoints. Among CGRs, we categorized focal amplifications if the maximum copy number exceeded the integer value of ploidy by more than four. Among focal amplifications, extrachromosomal DNA events were further identified if they corresponded to circular amplicons, as supported by the results of AmpliconArchitect^32^; otherwise, they were classified as complex amplifications.

### Timing of copy number gains

We estimated the timing of common copy number gains, as previously reported^19^, with modifications. First, for each SNV, we calculated the probability that it was attributed to clock-like signatures (SBS1 and SBS5/40)^33^. The probability was estimated based on the trinucleotide context of the SNV and the overall contribution of clock-like signatures across all SNVs in the tumour. Specifically, for SNVs in a given trinucleotide context, the probability was calculated as:

$$P\left(SB{S}_{clock}|context\right)=\frac{\mathop{\sum}\nolimits_{k\in \{1,5, 40\}}P\left(context{|}SB{S}_{k}\right)E(SB{S}_{k})}{\mathop{\sum }\nolimits_{k}P\left(context{|}SB{S}_{k}\right)E(SB{S}_{k})}$$

Here, $P\left({context|}{{SBS}}_{k}\right)$ denotes the probability of observing a given trinucleotide context under signature k, and $E\left({{SBS}}_{k}\right)$ represents the relative contribution of signature k in a given tumour.

In addition, we estimated the probability of each SNV being pre-amplificational, clonal, or subclonal based on the estimated copy numbers and VAFs. Specifically, (1) pre-amplificational mutations were assumed to have the major allele copy number, (2) clonal mutations were assumed to have copy numbers ranging from one to the major allele copy number, and (3) subclonal mutations were assumed to have copy numbers corresponding to the degree of subclonal expansion, which was estimated from the second peak of the cancer cell fraction distribution of SNVs in each tumour. The probability of an SNV being pre-amplificational was calculated as:

$$P\left({Preamp|TD},{AD},{totCN},{majCN}\right)=\frac{{Binom}({AD},{TD},{VA}{F}_{{totCN},{majCN},\rho })}{\mathop{\sum }\limits_{{CN}\in \{1,\ldots ,{majCN},{subCN}\}}{Binom}({AD},{TD},{VA}{F}_{{totCN},{CN},\rho })}$$

Here, *TD* and *AD* denote the total read depth and variant read depth, respectively, whereas *totCN, majCN* and *subCN* represent total copy number, major allele copy number, and degree of subclonal expansion, respectively. The expected VAF for a given total copy number and allele-specific copy number (*CN*) was calculated using the tumour purity *ρ* as*:*

$${{VAF}}_{{totCN},{CN},\rho }=\frac{\rho{\times}{CN}}{\rho{\times}{totCN}+\left(1-\rho \right)\times 2}$$

For each segment, we estimated the number of pre-amplificational, clonal and subclonal mutations attributed to clock-like signatures by summing the probabilities of SNVs within the segment that belonged to each category. These values were then adjusted based on the number of alleles to account for the increased exposure to mutations following copy number gains. Subsequently, the timing of the most recent common ancestor (MRCA), or the timing when all clonal mutations had accumulated, was estimated based on the densities of clonal and subclonal mutations and the patient’s age at diagnosis, assuming that each tumour maintained a consistent mutation rate and the number of detected subclonal mutations was proportional to the degree of subclonal expansion. The timing of the MRCA and the time interval between the MRCA and diagnosis were estimated as:

$${t}_{{MRCA}}=\frac{{d}_{{clonal}}}{{r}_{{clonal}}}$$

$${t}_{{dx}}-{t}_{{MRCA}}=\frac{{d}_{{subclonal}}}{{r}_{{subclonal}}}=\frac{{d}_{{subclonal}}}{{r}_{{clonal}}\times {subCN}}$$

Here, ${t}_{{MRCA}}$ and ${t}_{{dx}}$ represent the timing of the MRCA and the age at diagnosis, respectively. In addition, ${d}_{{clonal}}$ and ${d}_{{subclonal}}$ denote the densities of clonal and subclonal mutations, whereas ${r}_{{clonal}}$ and ${r}_{{subclonal}}$ denote the corresponding mutation accumulation rates.

Finally, we estimated the timing of copy number gains by converting the relative timing of the event, determined by the ratio of pre-amplificational to clonal mutations, into an absolute timing using the estimated timing of MRCA as a reference. For the timing analysis in the ICGC cohort, which lacks data on age at diagnosis, we estimated the timing of MRCA using the burden of clonal mutations and a median mutational rate derived from our cohort (4.4/Gb/year).

### Statistical analysis

All statistical analyses were performed using R (version 4.1.3). Chi-squared tests were used to assess differences in the frequency of categorical events, such as genomic alterations and metastatic progression, between groups. Pearson’s correlation test was used to examine associations between continuous variables. For comparisons among more than two groups, one-way ANOVA followed by Tukey’s post hoc test was applied. Relapse-free survival was defined as the period from the date of initial treatment to the date of metastatic detection (for patients who developed metastasis) or the date of last follow-up (for patients without metastasis). Log-rank tests were used to evaluate survival differences across genomic subgroups. All statistical tests were two-sided, and *P* values less than 0.05 were considered statistically significant.

## Results

### Mutational landscape of uveal melanomas

Our cohort included 40 individuals who underwent aspiration biopsy and were pathologically diagnosed with UM (Supplementary Table 1). The patients had a mean age of 58 years (range, 23–81), with a nearly equal distribution of sexes (19 females and 21 males). The proportions of patients who developed metastases and those who died were 30% (*n* = 12) and 23% (*n* = 9), respectively, with a median follow-up period of 3.5 years after treatment (range, 0.5–4.7). A strong predilection for liver metastasis was observed (*n* = 12; 100%), consistent with previous reports^1^, whereas the kidney, lung and bone were involved in 17% (*n* = 2), 8% (*n* = 1) and 8% (*n* = 1) of patients with metastasis, respectively (Supplementary Table 1).

**Fig. 1:**
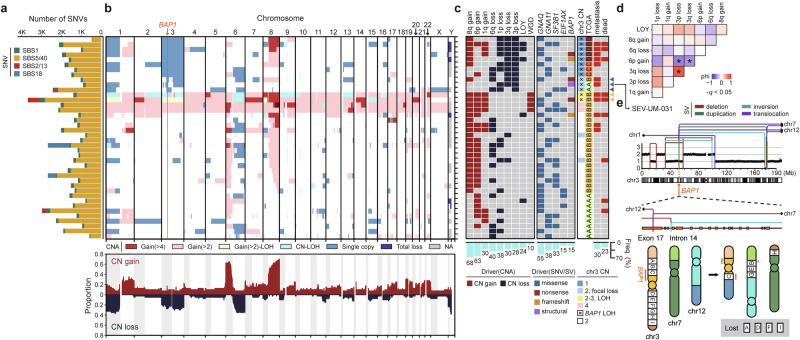
Mutational landscape of uveal melanomas. **a** Number of single-nucleotide variants (SNVs) in 40 uveal melanoma (UM) tumours. Coloured bars indicate the contributions of SBS signatures in each tumour. **b** Copy number alteration (CNA) landscapes in 40 UM tumours. The top panel shows coloured boxes indicating CNA status across genomic segments, with an orange line marking the genomic location of *BAP1* on chromosome 3p. The bottom panel shows the proportion of copy number gains and losses across genomic segments in the cohort. Grey and white backgrounds indicate the short and long arms of chromosomes, respectively. **c** Driver mutations, frequent CNAs, prognostic classification and clinical outcomes in 40 UM tumours. Blue and yellow triangles indicate tumours with *BAP1* loss of heterozygosity (LOH) due to segmental deletions and uniparental 3p, respectively. **d** Probabilities of co-occurrence and exclusiveness among frequent CNAs. Asterisks indicate *q* < 0.05. **e** Representative example of biallelic *BAP1* truncation by structural variations (SVs). CN, copy number; CN-LOH, copy-neutral LOH; Freq., frequency; LOY, loss of Y chromosome; NA, not applicable; WGD, whole-genome duplication.

We performed WGS on both tumours and matched blood samples (mean depth of 42.0 for tumours and 40.3 for blood) to identify somatic genomic variations, including SNVs, short indels, SVs and CNAs. The low burden of SNVs (median 1393; ranging from 462 to 3690), primarily attributable to clock-like mutational signatures (SBS1 and SBS5/40)^33^, was observed across all tumours, consistent with previous studies^15–17^ (Fig. 1a). Except for SBS18, a mutational signature linked to reactive oxygen species, mutations arising from external mutagens such as ultraviolet light were rare, as previously reported^16,17^. However, we identified evidence of APOBEC-induced mutagenesis in more than a quarter of tumours (*n* = 12; 30%; Supplementary Fig. 1). Among these, three exhibited a notable contribution of APOBEC-related signatures across the genome (SBS2/13; >3%), whereas the others harboured clustered APOBEC-associated mutations, known as *kataegis*^34^ (Supplementary Fig. 1).

Genome-wide copy number landscapes revealed prevalent arm-level CNAs, including gains in 8q (*n* = 27; 68%) and 6p (*n* = 25; 63%), as well as losses in 6q (*n* = 16; 40%), 1p (*n* = 15; 38%), 3q (*n* = 12; 30%) and 3p (*n* = 11; 28%; where the *BAP1* gene is located), as previously reported^35^ (Fig. 1b, c). In addition, 1q gain was also frequently observed in our cohort (*n* = 12; 30%), although it was not apparent in previous UM studies^11,35^. We also observed a relatively high prevalence of loss of the Y chromosome (LOY) (*n* = 5; 24% of male patients), in line with a previous report^36^, whereas loss of the X chromosome was less frequent (*n* = 3; 8%) in our cohort (Fig. 1b, c). Among these prevalent arm-level CNAs, 3p and 3q losses frequently occurred concomitantly, resulting in monosomy 3 (*φ* = 0.82, *q* = 4.7×10^−5^; Chi-squared test; Fig. 1b, d). Moreover, loss in 3p or 3q was mutually exclusive with 6p gain (3p loss and 6p gain, *φ* = -0.56, *q* = 0.019; 3q loss and 6p gain, *φ* = -0.51, *q* = 0.041; Chi-squared test; Fig. 1b, d). These relationships are consistent with the previous observation that monosomy 3 and 6p gain are associated with a poor and a favourable prognosis, respectively^10–12^. Despite the high frequency of arm-level CNAs, focal amplifications, which typically amplify a specific oncogene to a substantial copy number^32^, were rare in UM cancer genomes (*n* = 2; 5%; Supplementary Fig. 2).

A few specific genes were recurrently mutated in UMs (Fig. 1c). For example, *GNAQ* and *GNA11* harboured hotspot mutations (including *GNAQ* Q209P, R183Q, and Q209L, and *GNA11* Q209L), which were mutually exclusive and collectively observed in almost all tumour cases (*n* = 37; 93%), as previously reported^5^. Point mutations in the *BAP1* (*n* = 5; 13%), *SF3B1* (*n* = 13; 33%) and *EIF1AX* (*n* = 6; 15%) genes were also frequent.

Exploring whole-genome sequences enabled a more comprehensive evaluation of the driver mutations by integrating SVs. For example, one UM tumour (SEV-UM-031), which lacked *BAP1* point mutations, carried two loss-of-function SV hits in the *BAP1* gene: (1) loss of one allele owing to a 26 Mb segmental deletion and (2) disruption of the other allele by intragenic, truncating rearrangements, which fragmented the *BAP1* allele into three pieces (Fig. 1e). Similarly, we found two additional tumours with a complete loss of the *BAP1* gene locus owing to short segmental deletions (24 Mb and 38 Mb) in the absence of monosomy 3, as shown by focal copy losses at 3p encompassing *BAP1* (Fig. 1b, c). Our findings suggest that a substantial proportion of UMs harbour SV-associated *BAP1* disruption, which may not be readily captured by other assays, such as targeted sequencing and whole-exome sequencing.

### Metastasis-related mutations in uveal melanomas

**Fig. 2:**
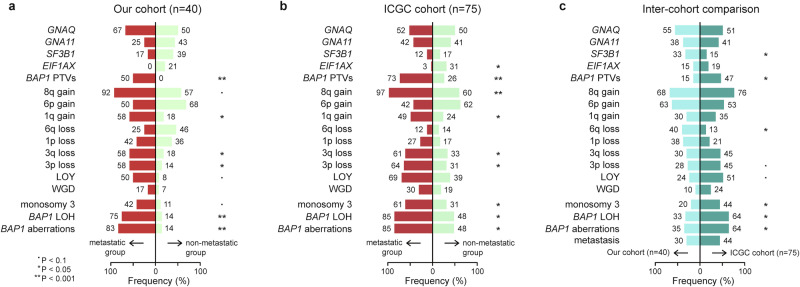
Metastasis-related mutations in uveal melanomas. **a**, **b** Frequency of genomic aberrations in patients with and without metastasis in our cohort (**a**) and in the International Cancer Genome Consortium (ICGC) cohort (**b**). *BAP1* protein-truncating variants (PTVs) include nonsense and frameshift mutations, essential splice-site variants and truncating structural variations affecting the *BAP1* coding sequence. *BAP1* loss of heterozygosity (LOH) includes monosomy 3, segmental deletion at the *BAP1* locus and uniparental 3p. *BAP1* aberrations encompass all genomic changes affecting the *BAP1* gene, including both *BAP1* PTVs and *BAP1* LOH. **c** Frequency of genomic aberrations in all patients within our cohort and the ICGC cohort. LOY, loss of Y chromosome; WGD, whole-genome duplication.

Some of the mutations were substantially enriched in tumours that developed metastasis, suggesting their contribution to metastatic progression in UM (Fig. 1c). All tumours with *BAP1* protein-truncating variants (PTVs), including nonsense mutations, frameshift mutations, essential splice-site variants and truncating SVs, exhibited metastasis in line with the previous observation^7^ (Fig. 1c). In addition, monosomy 3 was identified in eight (20%) tumours, more than half of which (*n* = 5, 63%) progressed to metastasis (Fig. 1b, c). Of note, among patients who experienced metastasis but did not carry monosomy 3 (*n* = 7), LOH at the *BAP1* locus was observed in more than half of the cases (*n* = 4; 57%), attributable to either SVs mentioned above (*n* = 2) or uniparental 3p (*n* = 2; Fig. 1b, c). Collectively, *BAP1* LOH, arising from monosomy 3, SV-associated copy number loss or uniparental 3p, was significantly more prevalent in the metastatic group than in the non-metastatic group (75% vs 14%, *P* = 7.0×10^−4^, Chi-squared test; Fig. 2a). Overall, *BAP1* alterations, encompassing all genomic changes affecting the *BAP1* gene, including PTVs and LOH, referred to as *BAP1* aberrations hereafter, were significantly enriched in the metastatic group (83% vs 14%, *P* = 1.3×10^−4^, Chi-squared test; Fig. 2a).

Among other alterations, 8q gain was more prevalent in the metastatic group (92% vs 57%, *P* = 0.077, Chi-squared test; Fig. 2a), whereas 6p gain was slightly more frequent in the non-metastatic group (50% vs 68%, *P* = 0.48, Chi-squared test; Fig. 2a). In addition, LOY was more enriched in the metastatic group (50% vs 8%, *P* = 0.092, Chi-squared test; Fig. 2a). These observations align with previous reports^10,36^; however, neither marker reached statistical significance in our cohort, presumably because of an inadequate cohort size. Instead, 1q gain, which has been largely underappreciated in previous studies^11,12,15,16^, was significantly more frequent in the metastatic group (58% vs 18%, *P* = 0.029, Chi-squared test; Fig. 2a). Its impact on metastatic progression was independent of both monosomy 3 and *BAP1* aberrations (Supplementary Fig. 3a–d), suggesting that 1q gain might play a role in metastatic progression and serve as a poor prognostic marker in UM.

To confirm the prognostic impact of *BAP1* aberrations and 1q gain, we validated their frequencies in an external cohort with 75 UM whole genomes studied by the ICGC^16^. Here, the frequency of *BAP1* aberrations in the metastatic group was 85%, whereas 48% of patients with no evidence of metastasis harboured *BAP1* aberrations, which aligns with our observation (*P* = 0.0020, Chi-squared test; Fig. 2b). In addition, 1q gain was more frequently identified in the metastatic group than in the non-metastatic group (49% vs 24%, *P* = 0.047, Chi-squared test; Fig. 2b).

Of note, despite the concordance of the metastasis-associated mutations, the frequencies of some driver mutations differed between the two cohorts. In particular, *BAP1* aberrations were significantly more frequent in the ICGC cohort than in our cohort (64% vs 35%, *P* = 0.0055, Chi-squared test; Fig. 2c). In line with this observation, the proportion of patients with metastasis was slightly higher in the ICGC cohort than in our cohort (44% vs 30%, *P* = 0.21; Fig. 2c), suggesting that characteristics of the cohorts may not be completely identical. We do not exclude the contribution of the inter-ethnic difference to the heterogeneous mutational landscapes and/or clinical courses, as our cohort and the ICGC cohort primarily consist of Asian and white patients, respectively.

### Refined genomic classification of uveal melanomas

**Fig. 3:**
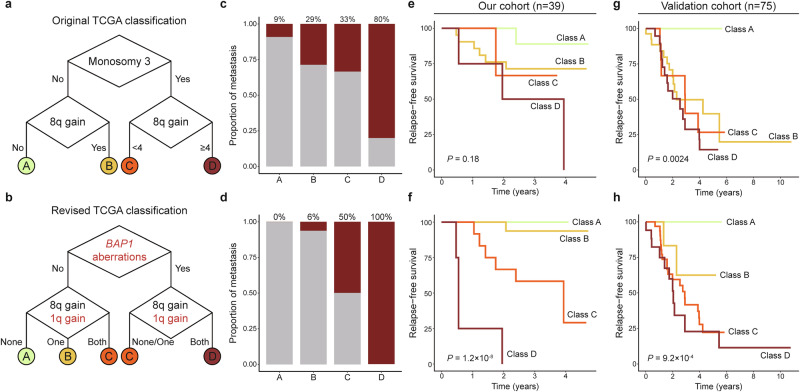
Refined genomic classification of uveal melanomas. **a**, **b** Classification criteria for the original The Cancer Genome Atlas (TCGA) classification (**a**) and the revised classification (**b**). The copies of 8q gains for classes C and D are equal to or greater than four. **c**, **d** Frequency of patients with metastasis in four classes of the original TCGA classification (**c**) and the revised classification (**d**). **e**, **f** Kaplan–Meier curves for relapse-free survival of 39 UM tumours in our cohort, stratified by the original TCGA classification (**e**) and the revised classification (**f**). One patient who presented with metastasis at the time of diagnosis was not included. **g**, **h** Kaplan–Meier curves for relapse-free survival of 75 UM tumours in the ICGC cohort^16^, stratified by the original TCGA classification (**g**) and the revised classification (**h**).

Based on the newly identified prognostic markers, we found that the UM classification criteria proposed by TCGA can be improved^11,12^. Specifically, the revised stratification includes (1) the use of *BAP1* aberrations instead of monosomy 3 to separate classes A/B from C/D and (2) the incorporation of 1q gain alongside 8q gain for further stratification into four classes (i.e. A, B, C and D; Fig. 3a, b). In addition, *BAP1* wild type tumours with both 8q and 1q gains were assigned to class C rather than class B, as their metastatic risk was comparable to that of tumours with *BAP1* aberrations (Supplementary Fig. 3e).

As expected, the revised genomic classification enhanced patient stratification by metastatic risk. Specifically, the new classification predicted UM metastasis more sensitively in our cohort, with metastatic proportions in classes A, B, C and D shifting from 9%, 29%, 33% and 80% to 0%, 6%, 50% and 100% respectively (Fig. 3c, d). Moreover, the relapse probabilities among the four groups showed clearer separation (*P* = 1.2×10^−8^; log-rank test) in the new classification than in the original TCGA classification (*P* = 0.18; log-rank test; Fig. 3e, f). The utility of the revised classification was validated using the independent ICGC cohort^16^, which showed better stratification of metastatic risk (*P* = 0.0024 for the original TCGA classification; *P* = 9.2×10^−4^ for the revised TCGA classification; log-rank test; Fig. 3g, h).

Another alternative classification (Supplementary Fig. 3f–h), which simply counts the number of poor prognostic biomarkers present in each tumour, showed prognostic performance comparable to that of our revised genomic classification. Notably, this approach does not assign greater weight to *BAP1* aberrations than the original or revised TCGA classification. We anticipate that a larger cohort will be required to robustly distinguish the performance of the revised and alternative classification schemes.

### Timing of prognostic copy number gains in uveal melanomas

**Fig. 4:**
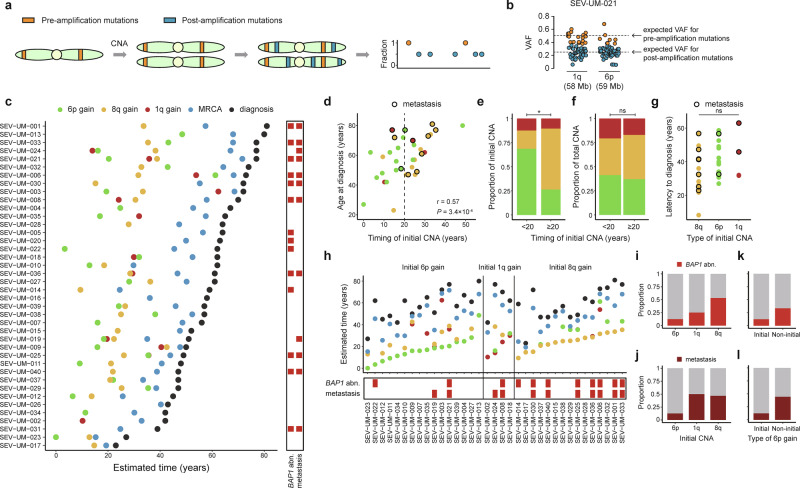
Timing of prognostic copy number alterations in uveal melanomas. **a** Diagram illustrating the principles of mutation timing analysis. **b** Representative example of mutation timing analysis. The gain in 6p with fewer co-amplified single-nucleotide variants (SNVs) is estimated to precede the gain in 1q with more co-amplified SNVs. **c** Estimated timing of prevalent copy number gains and most recent common ancestor (MRCA) presented with the age at diagnosis, the presence of *BAP1* aberrations and metastasis in 40 uveal melanoma (UM) tumours. **d** Relationship between the timing of initial copy number alterations (CNAs) and age at diagnosis in 35 UM tumours. Five tumours without prevalent copy number gains were not included. **e**, **f** Proportion of initial CNAs (**e**) and total CNAs (**f**) between tumours with initial CNAs estimated to occur before (*n* = 16) and after the age of 20 (*n* = 19). **g** Estimated latency from initial CNA to diagnosis across different types of initial CNAs in 35 UM tumours. **h** Estimated timing of prevalent copy number gains and MRCA presented with the age at diagnosis, the presence of *BAP1* aberrations and metastasis across different types of initial CNAs in 35 UM tumours. **i**, **j** Proportion of *BAP1* aberrations (**i**) and metastasis (**j**) among tumours with different types of initial CNAs (initial 6p gains, *n* = 16; initial 1q gains, *n* = 4; initial 8q gains, *n* = 15). **k**, **l** Proportion of *BAP1* aberrations (**k**) and metastasis (**l**) among tumours with initial 6p gains (*n* = 16) and non-initial 6p gains (*n* = 9). *BAP1* abn., *BAP1* aberrations; ns, not significant; VAF, variant allele fraction.

After observing the impact of CNAs on metastatic potential, we investigated the mutational trajectory of tumour cell lineages during tumorigenesis, focusing on the acquisition timing of prognosis-related copy number gains, such as 6p, 8q and 1q gains. The timing of copy number gains can be estimated based on the clock-like property of co-amplified SNVs within an amplicon, where an amplification event in the late stage of a lifetime shows a higher density of co-amplified SNVs that predated the amplification event^18,19^ (Fig. 4a). For example, in SEV-UM-021, we concluded that the 1q gain occurred later than the 6p gain, as we found a substantially higher density of co-amplified SNVs on 1q (17 vs 8 co-amplified SNVs for 58 Mb-sized 1q and 59 Mb-sized 6p amplicons, respectively; Fig. 4b). Under an assumption of a consistent rate of endogenous mutational processes over a lifetime^33^, we converted the relative amplification timing into an absolute physical age (Materials and Methods).

Interestingly, our timing analysis revealed that the first copy number gains of the three chromosome arms in each tumour often occurred during the early stages of life, with an average age of 20.7 years (Fig. 4c), as observed in other cancer types^18,19^. On the median, an initial CNA event emerged several decades (median 36.1 years) before clinical diagnosis (Fig. 4c). Despite the substantial variation in the latency from an initial copy number gain to diagnosis among patients (from 8 (SEV-UM-017) to 63 years (SEV-UM-024)), there was a clear correlation between the timing of initial CNAs and the absolute age at diagnosis (*r* = 0.57, *P* = 3.4×10^−4^, Pearson’s test; Fig. 4d). These findings suggest that genomic instability starts early in life in the somatic lineage to cancer, and the evolution into fully neoplastic cells mostly takes longer than a decade in UM tumorigenesis, potentially for the acquisition of additional factors.

Of note, the dominant type of initial CNAs shifted with the timing of the events (Fig. 4d, e). Specifically, the initial CNAs estimated to occur before the age of 20 were predominantly 6p gains (*n* = 11, 69%), whereas those estimated to arise after the age of 20 were primarily 8q gains (*n* = 12, 63%, *P* = 0.024, Chi-squared test; Fig. 4d, e). This difference was not driven by an overall increase in CNA frequency, as the proportion of total CNAs remained consistent between the two tumour groups (*P* = 0.82, Chi-squared test; Fig. 4f).

We explored how the type of initial CNAs affects the characteristics of UM tumours. The latency from the initial CNAs to clinical diagnosis did not show significant differences across the types of initial CNAs, although the median interval was slightly shorter for 8q gains (32.5 years) compared with 6p gains (38.7 years) and 1q gains (39.0 years, *P* = 0.22; one-way ANOVA test; Fig. 4g). Our findings suggest that UM development generally spans 30–40 years, regardless of the type of initial CNA.

Interestingly, we further observed that initial CNA events were associated with the molecular characteristics and metastatic potential of UM (Fig. 4h). For example, tumours with initial 6p gains showed a lower frequency of *BAP1* aberrations compared with those with initial 1q gains and 8q gains (13% vs 25% vs 53%, *P* = 0.048, Chi-squared test; Fig. 4i). As a result, the metastatic risk was lowest in tumours with initial 6p gains (13% vs 50% vs 47% for initial 6p, 1q and 8q gains, respectively, *P* = 0.086, Chi-squared test; Fig. 4j). Although not reaching statistical significance, *BAP1* aberrations were less frequent in tumours with initial 6p gains than in those with non-initial 6p gains (13% vs 33%, *P* = 0.47, Chi-squared test; Fig. 4k), which corresponded to differences in metastatic risk (13% vs 44%, *P* = 0.19, Chi-squared test; Fig. 4l). Overall, our findings suggest that the order of chromosomal gains might play a crucial role in shaping the characteristics of UM tumours. Although the mechanism is uncertain, we speculate that the first chromosomal gain can influence the trajectory of subsequent mutations for neoplastic transformation, as evidenced by the mutual exclusivity between the 6p gain and *BAP1* aberrations described above.

To validate our findings, we conducted the timing analysis in the ICGC cohort despite the lack of data on age at tumour diagnosis. Using the median mutational rate from our cohort (4.4/Gb/year), we revealed that the initial CNA events primarily occurred during the early stages of life (mean age of 23.6) and decades before the emergence of MRCA of the tumour (mean 26.9 years; Supplementary Fig. 4a, b), supporting the findings from our cohort. In contrast to our cohort, 8q gains were the predominant earliest CNAs regardless of the acquisition timing (before the age of 20, *n* = 13, 50%; after the age of 20, *n* = 29, 69%; Supplementary Fig. 4c, d). The latency from initial CNAs to MRCA was not significantly different across the types of initial CNAs, consistent with the findings in our cohort (*P* = 0.25; one-way ANOVA test; Supplementary Fig. 4e). Of note, the impact of initial CNAs on molecular characteristics and metastatic risk was also observed (Supplementary Fig. 4f): tumours with initial 6p gains carried *BAP1* aberrations less frequently (28% for initial 6p gains vs 63% for initial 1q gains vs 86% for initial 8q gains; *P* = 6.0 ×10^−5^, Chi-squared test; Supplementary Fig. 4g), which resulted in lower rates of metastatic progression (22% vs 38% vs 60% for initial 6p, 1q and 8q gains, respectively; *P* = 0.025, Chi-squared test; Supplementary Fig. 4h). The impact was also detected between tumours with initial and non-initial 6p gains regarding *BAP1* aberration frequency (28% vs 64%, *P* = 0.052, Chi-squared test; Supplementary Fig. 4i) and metastatic risk (22% vs 46%; *P* = 0.23, Chi-squared test; Supplementary Fig. 4j), further supporting our speculation on the importance of the order of mutations in determining the tumour’s characteristics in UM.

## Discussion

In this study, we comprehensively illustrated the mutational landscapes of UM using WGS, focusing on genomic aberrations related to metastasis. Our findings revealed that *BAP1* aberrations and 1q gain are independent and highly significant poor prognostic markers associated with metastatic progression in UM. Integrating these two markers into a genomic classification system enhanced prognosis prediction. Furthermore, timing analysis showed that prevalent copy number gains, including 6p, 8q and 1q gains, frequently appeared during the early decades of life and the initial stages of tumour development, contributing to the early determination of metastatic potentials in UM tumours.

Monosomy 3 is regarded as the most significant indicator of poor prognosis in UM^6,11,12,37^. Here, we observed recurrent CNAs, including focal losses and copy-neutral LOHs, spanning the *BAP1* gene locus on 3p, particularly in patients with metastasis. It suggests that the primary changes associated with monosomy 3 could involve the loss of one functional *BAP1* allele, leading us to introduce the concept of *BAP1* aberrations, encompassing point mutations, SVs, CNAs and LOHs, that overall result in loss-of-function alterations in the *BAP1* gene. Indeed, *BAP1* aberrations were enriched in the metastatic group and stratified patients by metastatic risk more effectively than monosomy 3. This emphasizes the ability of WGS to sensitively detect genomic alterations, which is essential for risk stratification and clinical management of UM patients. Promoter deletions can be a type of *BAP1* aberrations^38^, although none was observed in our cohort. Of note, *BAP1* aberrations are not always biallelic, despite the role of *BAP1* as a tumour suppressor. Considering the metastatic rates in biallelic (*n* = 5) and monoallelic (*n* = 9) *BAP1* aberrations are 100% and 56%, respectively (Fig. 1c), even monoallelic *BAP1* aberrations can contribute to metastatic progression, possibly because of *BAP1* haploinsufficiency^39^ or suppression of the remaining *BAP1* allele through promoter methylation^40^.

We also proposed 1q gain as a metastasis-related genomic alteration, in addition to *BAP1* aberrations. Previous studies described the enrichment of 1q gain in metastatic tumours compared with primary tumours^15,35^. However, 1q gain has generally been underappreciated and not utilized to estimate the metastatic potential of tumours^11,12^. The refined genomic classification, which incorporates 1q gain into its criteria, demonstrated a more effective separation between patients with and without metastasis than the previous TCGA classification. Still, it remains unclear which gene constitutes the key driver within 1q and how large-scale copy number gains contribute to tumour formation and metastatic progression in melanocytes. Further experimental validation is necessary to establish the mechanistic role of 1q gain in metastatic progression. The relative and potentially interacting contributions of *BAP1* aberrations and 1q gain to metastatic progression also require further investigation. Accordingly, we did not adopt an alternative genomic classification that assigns equal weights to *BAP1* aberrations, 8q gain and 1q gain, despite its comparable performance (Supplementary Fig. 3f–h), given the long-standing evidence that *BAP1* mutations and monosomy 3 are major determinants of metastatic risk in UM^6,7,11,12,37^.

In addition, we gained valuable insights into the genomic evolution of UM by estimating the timing of prevalent copy number gains in chronological years. First, the earliest chromosomal aberrations in UM are estimated to occur during adolescence and early adulthood in most cases, with some traced back to even a few years after birth. This observation is consistent with previous reports on other types of cancers^18,19^, suggesting that the initial clone with large-scale CNAs, which will eventually develop into cancer, emerges in the early stages of life in various types of cancers. Indeed, recent studies demonstrated the pervasive mosaic chromosomal alterations in normal human cells^41^. From this perspective, our study emphasizes the importance of a comprehensive investigation into somatic mosaicism in the human body, which potentially offers the opportunity for the early detection of and intervention in cancer.

Furthermore, we observed that metastasis-related CNAs were frequently estimated to occur decades before diagnosis, even preceding the MRCA of primary tumours. As up to 50% of patients experience metastatic relapse despite successful local treatment, it has been hypothesized that UM cancer cells often metastasize during the tumorigenesis period. Previous research also proposed early metastatic dissemination based on targeted sequencing on primary and matched metastatic UM^42^. Our findings support this perspective and further enhance the understanding of the UM evolution by comparing the temporal sequence of CNAs, MRCA and clinical diagnosis in chronological years.

We detected a mutually exclusive occurrence between 6p gain and *BAP1* aberrations, consistent with previous studies that describe the inverse relationship between 6p gain and monosomy 3^43^ and the opposite prognostic implications of these two CNAs^10–12^. Of note, this relationship was more prominent in tumours with an initial CNA of 6p gain, leading to differences in the rates of *BAP1* aberrations and metastatic risk even between patients with initial and non-initial 6p gains. Our findings suggest that 6p gain has a “protective” effect on metastatic progression by blocking the occurrence of *BAP1* aberrations. We speculate that *BAP1* aberrations can offer no survival benefits or even disadvantages in melanocytes when concomitantly occurring with 6p gain. However, further studies are warranted to fully elucidate the underlying mechanism of mutual exclusiveness between 6p gain and *BAP1* aberrations.

Interestingly, the dominant type of initial CNAs differs across the age of occurrence. Specifically, 6p gain and 8q gain are predominant copy number gains before and after the age of 20, respectively. It is consistent with previous reports on the relevance of young age to the better prognosis in UM^44^. We speculate that younger patients can have a better prognosis because the initial CNAs in their tumours are more likely to be 6p gains, which can provide a protective effect against *BAP1* aberrations and metastatic progression.

In our all-Korean cohort, the metastatic rate and the incidence of *BAP1* aberrations were lower than in the ICGC cohort, which is predominantly composed of white people. Previous studies have reported that Asian patients tend to be diagnosed at a younger age with larger tumours, yet their prognosis appears to be better, or at least not worse, than that of white people^45–47^. Further studies are needed to determine whether the lower frequency of poor prognostic genetic aberrations is a characteristic specific to Asian patients with UM.

This study has several limitations. First, a subset of tumours exhibited low tumour purity, which encouraged us to rescue known driver mutations for tumour characterization. Second, the low incidence of UM in Asian populations resulted in a relatively smaller sample size, which we addressed by validating key findings in a larger, independent cohort. In addition, inter-cohort differences, possibly derived from population-specific characteristics, warrant further investigation in larger, globally representative cohorts. Finally, although this study primarily focuses on genomic analyses, additional functional studies will be needed to fully elucidate the biological impact of specific genomic aberrations on metastatic progression.

In summary, we identified *BAP1* aberrations and 1q gain as novel poor prognostic markers and proposed a refined genomic classification system that better predicts the metastatic risk in UM. We also estimated the timing of prevalent and prognosis-related copy number gains in chronological years by analysing WGS data from primary UM. Our analysis suggests that initial clones with driver genomic alterations usually emerge early in life and take several decades to eventually develop into cancer cells, and their metastatic potentials are generally determined during the initial stages of tumour development. Overall, our study offers insights into the genomic evolution of UM, laying the groundwork for early detection and prognostication.

**Supplementary Information** accompanies the manuscript on the Experimental & Molecular Medicine website (http://www.nature.com/emm/).

## Supplementary information

Supplementary Information

Supplementary Table 1

## Data Availability

Whole-genome sequencing data are deposited in the European Genome–phenome Archive under accession no. EGAD50000002292 and are available for general research use. In-house scripts for analyses are available on GitHub (https://github.com/changhnam/UM_WGS).
