# Supplementary material for: Metastasis-related genomic aberrations and evolutionary trajectory in uveal melanoma
Source: Exp Mol Med. 2026 Jun 17;58(6):2024–32. doi: 10.1038/s12276-026-01750-y (PMC13324459; doi:10.1038/s12276-026-01750-y)
Supplement: Supplementary file 1 — Supplementary Information [file 12276_2026_1750_MOESM1_ESM.pdf]

Supplementary Information for

Metastasis-related genomic aberrations and evolutionary trajectory in uveal melanoma

Chang Hyun Nam, Yong Joon Kim, Jeonghwan Youk, Hyo Song Park, Young Seok Ju, Christopher Seungkyu Lee

Supplementary Figures

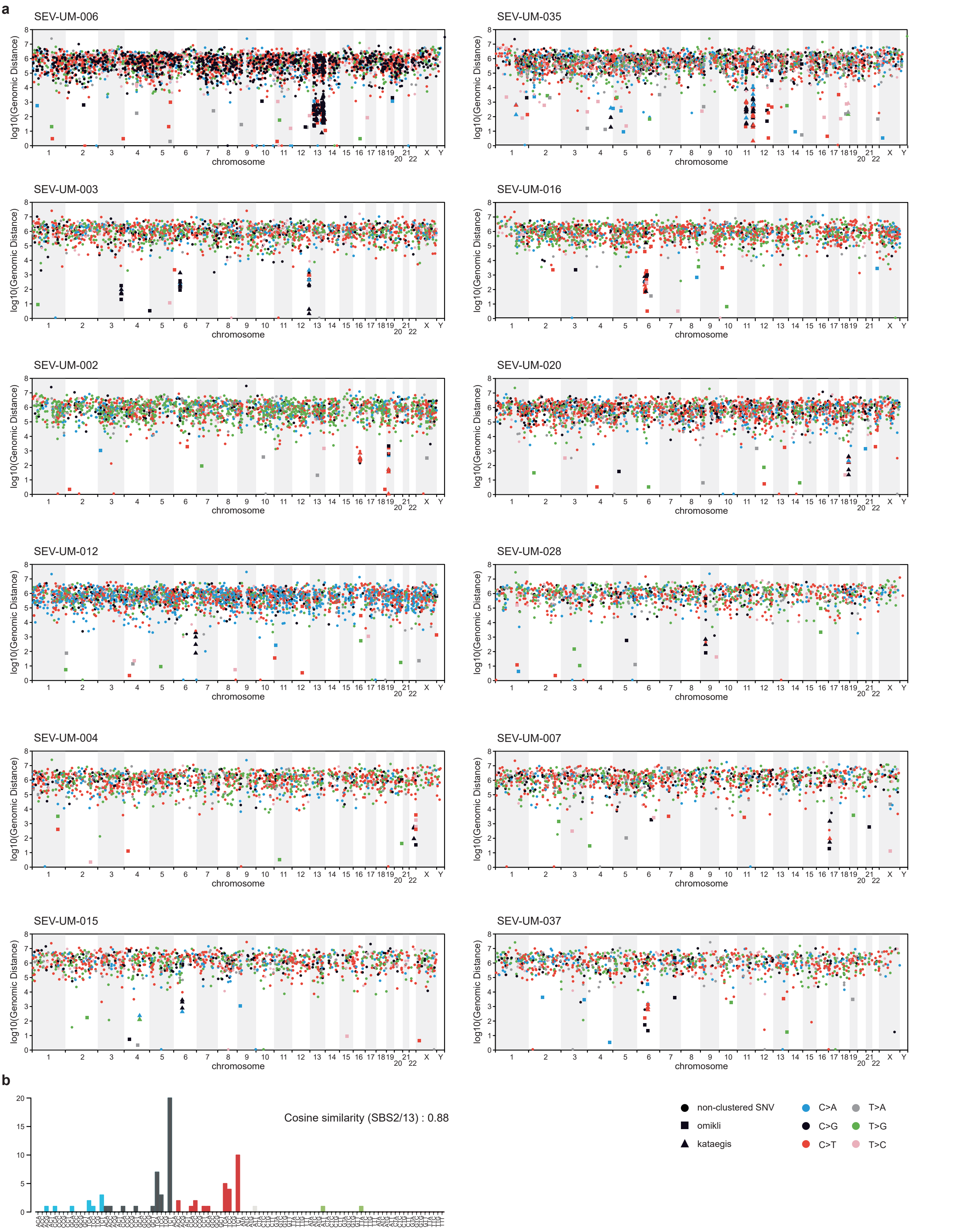

Supplementary Figure 1. Evidence of APOBEC-induced mutagenesis in uveal melanomas.
a, Genomic distances between adjacent SNVs in 12 UM tumors with clustered C>T and C>G mutations, supporting APOBEC-induced mutagenesis. **b**, Mutational profile of clustered SNVs identified in nine UM tumors with no significant contribution from APOBEC signatures (SBS2/13). The profile closely resembled SBS2/13, suggesting that the mutations likely originated from APOBEC activity.

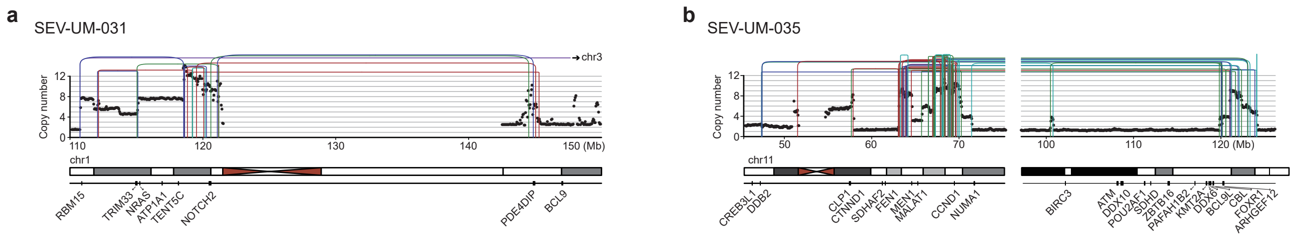

Supplementary Figure 2. Complex genomic rearrangements in uveal melanomas.
a,b, Two examples of complex genomic rearrangements that amplify regions encompassing the *NOTCH2* gene (**a**) and *CCND1* gene (**b**), respectively.

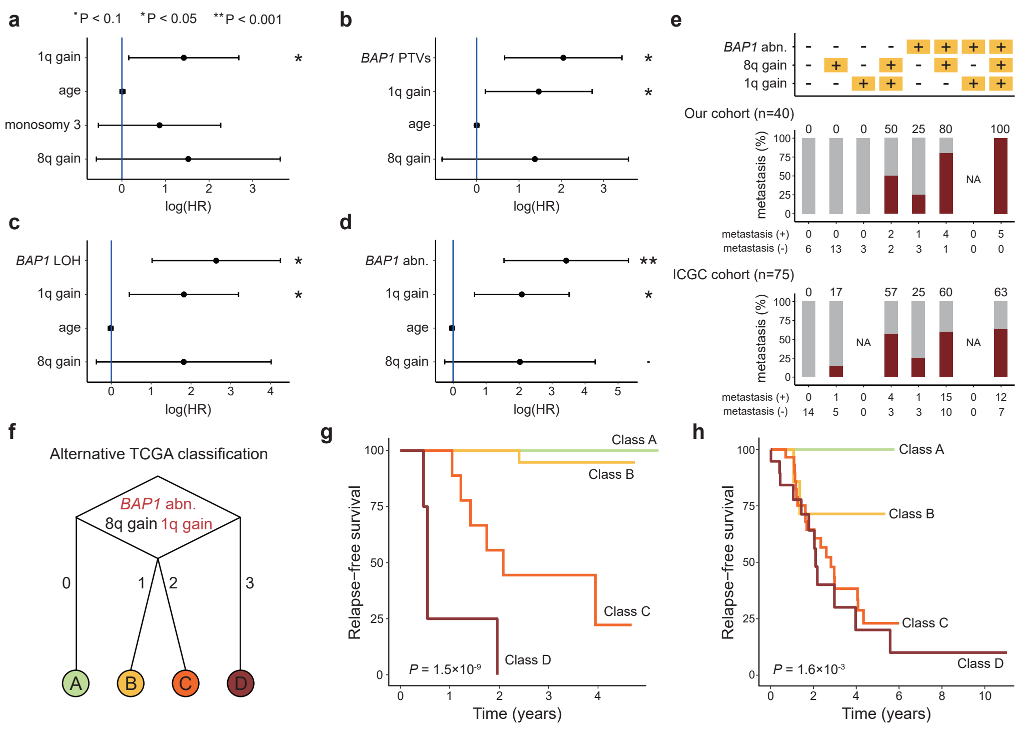

Supplementary Figure 3. Unfavorable prognostic markers in uveal melanomas.
a-d, Multivariate Cox regression analysis considering age, 8q gain, and 1q gain alongside monosomy 3 (**a**), *BAP1* PTVs (**b**), *BAP1* LOH (**c**), and *BAP1* aberrations (**d**), respectively. **e**, Proportion of metastasis in tumors with different mutational statuses, including *BAP1* aberrations, 8q gains, and 1q gains (top), in our cohort (middle) and in the ICGC cohort (bottom). **f,** Classification criteria for the alternative TCGA classification. **g,h,** Kaplan-Meier curves for relapse-free survival in 39 UM tumors from our cohort (**g**) and 75 UM tumors from the ICGC cohort (**h**). In our cohort, one patient who presented with metastasis at the time of diagnosis was not included. HR, hazard ratio; PTVs, protein-truncating variants; LOH, loss of heterozygosity; *BAP1* abn., *BAP1* aberrations.

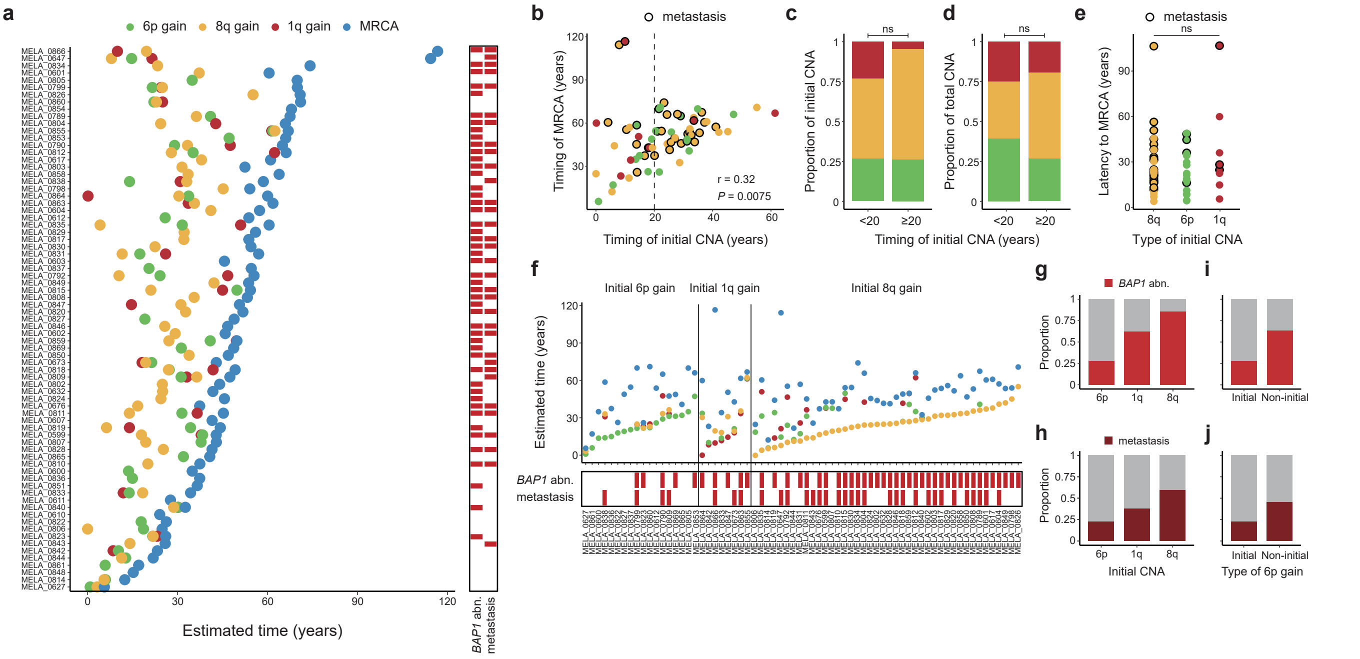

Supplementary Figure 4. Timing of prognostic CNAs in uveal melanomas in the ICGC cohort. a, Estimated timing of prevalent copy number gains and MRCA presented with the presence of *BAP1* aberrations and metastasis in 75 UM tumors. **b**, Relationship between the timing of initial CNAs and MRCA in 68 UM tumors. Tumors without prevalent copy number gains (n=7) were not included. **c**,**d**, Proportion of initial CNAs (**c**) and total CNAs (**d**) between tumors with initial CNAs estimated to occur before age 20 (n=26) and after age 20 (n=42). **e**, Estimated latency from initial CNA to MRCA across different types of initial CNAs in 68 UM tumors. **f**, Estimated timing of prevalent copy number gains and MRCA presented with the presence of *BAP1* aberrations and metastasis across different types of initial CNAs in 68 UM tumors. **g**,**h**, Proportion of *BAP1* aberrations (**g**) and metastasis (**h**) among tumors with different types of initial CNAs. (initial 6p gains, n=18; initial 1q gains, n=8; initial 8q gains, n=42). **i**,**j**, Proportion of *BAP1* aberrations (**i**) and metastasis (**j**) among tumors with initial 6p gains (n=18) and non-initial 6p gains (n=22). MRCA, most recent common ancestor; *BAP1* abn., *BAP1* aberrations; CNA, copy number alteration; ns, not significant.

Supplementary Table Legends

Supplementary Table 1. Clinical and mutational characteristics of 40 primary uveal melanomas. The table provides comprehensive information on each tumor, including patient age at diagnosis, sex, clinical assessment, treatment history, survival and metastasis outcomes, and mutational characteristics such as copy number alterations and driver mutations.
